# *N*-(4-Bromo­phen­yl)-4-nitro­benzamide

**DOI:** 10.1107/S1600536811000122

**Published:** 2011-01-08

**Authors:** Sohail Saeed, Jerry P. Jasinski, Ray J. Butcher

**Affiliations:** a Department of Chemistry, Research Complex, Allama Iqbal Open University, Islamabad 44000, Pakistan; b Department of Chemistry, Keene State College, 229 Main Street, Keene, NH 03435-2001, USA; c Department of Chemistry, Howard University, 525 College Street NW, Washington, DC 20059, USA

## Abstract

In the title compound, C_13_H_9_BrN_2_O_3_, the dihedral angle between the mean planes of the two benzene rings is 3.6 (7)°. The amide group is twisted by 28.1 (6) and 31.8 (3)° from the mean planes of the 4-bromo­phenyl and 4-nitro­benzene rings, respectively. The crystal packing features weak inter­molecular N—H⋯O and C—H⋯O hydrogen bonds resulting in a three-dimensional network.

## Related literature

For the anti­microbial activity of amides, see: Priya *et al.* (2005 ▶). For the use of amides in supra­molecular chemical anion sensor technology, see: Jagessar & Rampersaud (2007 ▶). For a related structure, see: Gowda *et al.* (2008 ▶);
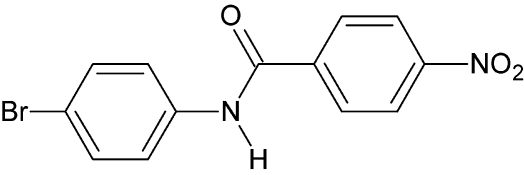


## Experimental

### 

#### Crystal data

C_13_H_9_BrN_2_O_3_
*M* *_r_* = 321.13
Monoclinic, 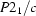

*a* = 4.57903 (6) Å
*b* = 12.92579 (15) Å
*c* = 20.5614 (3) Å
β = 96.0333 (11)°
*V* = 1210.24 (3) Å^3^
*Z* = 4
Cu *K*α radiation
μ = 4.70 mm^−1^
*T* = 123 K
0.48 × 0.12 × 0.07 mm

#### Data collection

Oxford Diffraction Xcalibur Ruby Gemini diffractometer
Absorption correction: multi-scan (*CrysAlis RED*; Oxford Diffraction, 2007 ▶) *T*_min_ = 0.485, *T*_max_ = 1.000
8049 measured reflections
2434 independent reflections
2329 reflections with *I* > 2σ(*I*)
*R*_int_ = 0.025

#### Refinement

*R*[*F* ^2^ > 2σ(*F* ^2^)] = 0.027
*wR*(*F* ^2^) = 0.075
*S* = 1.06
2434 reflections
172 parameters
H-atom parameters constrained
Δρ_max_ = 0.52 e Å^−3^
Δρ_min_ = −0.29 e Å^−3^

### 

Data collection: *CrysAlis PRO* (Oxford Diffraction, 2007 ▶); cell refinement: *CrysAlis RED* (Oxford Diffraction, 2007 ▶); data reduction: *CrysAlis RED*; program(s) used to solve structure: *SHELXS97* (Sheldrick, 2008 ▶); program(s) used to refine structure: *SHELXL97* (Sheldrick, 2008 ▶); molecular graphics: *SHELXTL* (Sheldrick, 2008 ▶); software used to prepare material for publication: *PLATON* (Spek, 2009 ▶).

## Supplementary Material

Crystal structure: contains datablocks global, I. DOI: 10.1107/S1600536811000122/pv2370sup1.cif

Structure factors: contains datablocks I. DOI: 10.1107/S1600536811000122/pv2370Isup2.hkl

Additional supplementary materials:  crystallographic information; 3D view; checkCIF report

## Figures and Tables

**Table 1:** Hydrogen-bond geometry (Å, °)

*D*—H⋯*A*	*D*—H	H⋯*A*	*D*⋯*A*	*D*—H⋯*A*
N1—H1*A*⋯O1^i^	0.88	2.33	3.0026 (18)	133
N1—H1*A*⋯O2^ii^	0.88	2.59	3.284 (2)	136
C3—H3*A*⋯O1^iii^	0.95	2.45	3.284 (2)	146
C5—H5*A*⋯O3^iv^	0.95	2.52	3.447 (2)	166
C6—H6*A*⋯O2^ii^	0.95	2.49	3.354 (2)	151
C9—H9*A*⋯O2^ii^	0.95	2.48	3.397 (2)	162

Symmetry codes: (i) 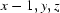; (ii) 
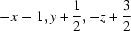
; (iii) 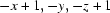; (iv) 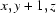.

**Fig. 1.**
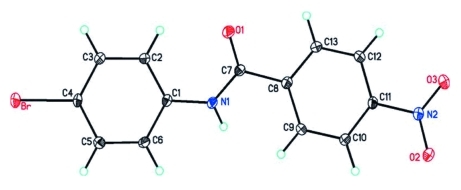
Molecular structure of the title compound showing the atom labeling scheme and 50% probability displacement ellipsoids.

**Fig. 2.**
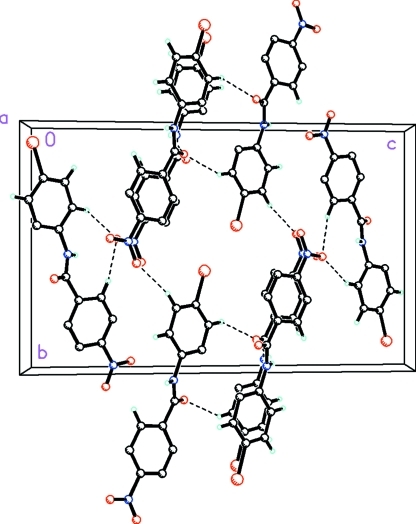
Packing diagram of the title compound viewed down the a axis; hydrogen bonds are indicated by dashed lines indicate and H-atoms not involved in hydrogen bonding have been excluded for clarity.

## supplementary crystallographic information

### Comment

Amides are known to play a pivitol role in molecular recognition, being important components in supramolecular chemical anion sensors technology (Jagessar & Rampersaud, 2007). Moreover, amides have also been reported as antimicrobial agents (Priya *et al.*, 2005). The structure of the title compound has been determined to explore the effect of substituents on the structure of benzanilides.

In the title compound (Fig. 1), the dihedral angle between the mean planes of the two benzene rings is 3.6 (7)°. The amide group is twisted by 28.1 (6) and 31.8 (3)° from the mean planes of the 4-bromophenyl and 4-nitrobenzene rings. The bond distances and angles in the title compound agree well with the corresponding bond distances and angles reported for a closely related compound (Gowda *et al.*, 2008). The crystal packing of the title compound is stabilized by weak N—H···O and C—H···O intermolecular hydrogen bonds which results in a hydrogen bonded 3-D network (Fig. 2).

### Experimental

A solution of 4-nitrobenzoyl chloride (0.01 mol) and 4-bromoaniline (0.01 mol) in anhydrous acetone was refluxed for 4 h. After completion of the reaction, the crude solid product was filtered, washed with water and purified by re-crystallization from ethyl acetate.

### Refinement

The N–H atom length was set to 0.88Å (NH) and the H atom refined isotropically.

The H atoms were placed in their calculated positions with N—H = 0.88 and C—H = 0.95° A and refined using the riding model with isotropic displacement parameters set to 1.2 times *U*_eq_ of the parent atoms.

### Crystal data

**Table:**

C_13_H_9_BrN_2_O_3_	*F*(000) = 640
*M**_r_* = 321.13	*D*_x_ = 1.762 Mg m^−^^3^
Monoclinic, *P*2_1_/*c*	Cu *K*α radiation, λ = 1.54184 Å
Hall symbol: -P 2ybc	Cell parameters from 7736 reflections
*a* = 4.57903 (6) Å	θ = 5.5–73.9°
*b* = 12.92579 (15) Å	µ = 4.70 mm^−^^1^
*c* = 20.5614 (3) Å	*T* = 123 K
β = 96.0333 (11)°	Needle, colorless
*V* = 1210.24 (3) Å^3^	0.48 × 0.12 × 0.07 mm
*Z* = 4	

### Data collection

**Table:**

Oxford Diffraction Xcalibur Ruby Gemini diffractometer	2434 independent reflections
Radiation source: Enhance (Cu) X-ray Source	2329 reflections with *I* > 2σ(*I*)
graphite	*R*_int_ = 0.025
Detector resolution: 10.5081 pixels mm^-1^	θ_max_ = 74.0°, θ_min_ = 5.5°
ω scans	*h* = −5→5
Absorption correction: multi-scan (*CrysAlis RED*; Oxford Diffraction, 2007)	*k* = −15→15
*T*_min_ = 0.485, *T*_max_ = 1.000	*l* = −20→25
8049 measured reflections	

### Refinement

**Table:**

Refinement on *F*^2^	Primary atom site location: structure-invariant direct methods
Least-squares matrix: full	Secondary atom site location: difference Fourier map
*R*[*F*^2^ > 2σ(*F*^2^)] = 0.027	Hydrogen site location: inferred from neighbouring sites
*wR*(*F*^2^) = 0.075	H-atom parameters constrained
*S* = 1.06	*w* = 1/[σ^2^(*F*_o_^2^) + (0.0496*P*)^2^ + 0.6292*P*] where *P* = (*F*_o_^2^ + 2*F*_c_^2^)/3
2434 reflections	(Δ/σ)_max_ = 0.002
172 parameters	Δρ_max_ = 0.52 e Å^−^^3^
0 restraints	Δρ_min_ = −0.29 e Å^−^^3^

### Special details

**Table:**

Geometry. All e.s.d.'s (except the e.s.d. in the dihedral angle between two l.s. planes) are estimated using the full covariance matrix. The cell e.s.d.'s are taken into account individually in the estimation of e.s.d.'s in distances, angles and torsion angles; correlations between e.s.d.'s in cell parameters are only used when they are defined by crystal symmetry. An approximate (isotropic) treatment of cell e.s.d.'s is used for estimating e.s.d.'s involving l.s. planes.
Refinement. Refinement of *F*^2^ against ALL reflections. The weighted *R*-factor *wR* and goodness of fit *S* are based on *F*^2^, conventional *R*-factors *R* are based on *F*, with *F* set to zero for negative *F*^2^. The threshold expression of *F*^2^ > σ(*F*^2^) is used only for calculating *R*-factors(gt) *etc*. and is not relevant to the choice of reflections for refinement. *R*-factors based on *F*^2^ are statistically about twice as large as those based on *F*, and *R*- factors based on ALL data will be even larger.

### Fractional atomic coordinates and isotropic or equivalent isotropic displacement parameters (Å^2^)

**Table:**

	*x*	*y*	*z*	*U*_iso_*/*U*_eq_	
Br	0.32019 (4)	0.406567 (14)	0.541374 (10)	0.02940 (10)	
O1	0.3492 (3)	−0.11877 (10)	0.59423 (6)	0.0247 (3)	
O2	−0.5374 (3)	−0.46316 (11)	0.76725 (7)	0.0345 (3)	
O3	−0.2924 (4)	−0.55958 (11)	0.70717 (8)	0.0377 (4)	
N1	−0.0486 (3)	−0.02526 (11)	0.61758 (7)	0.0201 (3)	
H1A	−0.2223	−0.0300	0.6320	0.024*	
N2	−0.3666 (3)	−0.47512 (12)	0.72579 (7)	0.0231 (3)	
C1	0.0405 (3)	0.07385 (13)	0.59764 (8)	0.0182 (3)	
C2	0.2236 (4)	0.08785 (13)	0.54822 (9)	0.0201 (3)	
H2A	0.2927	0.0295	0.5262	0.024*	
C3	0.3048 (4)	0.18693 (15)	0.53110 (8)	0.0219 (3)	
H3A	0.4303	0.1968	0.4976	0.026*	
C4	0.2009 (4)	0.27132 (13)	0.56336 (8)	0.0198 (3)	
C5	0.0130 (4)	0.25951 (14)	0.61141 (9)	0.0239 (4)	
H5A	−0.0597	0.3182	0.6324	0.029*	
C6	−0.0673 (4)	0.16009 (14)	0.62825 (9)	0.0230 (3)	
H6A	−0.1968	0.1508	0.6610	0.028*	
C7	0.1117 (3)	−0.11336 (13)	0.61622 (8)	0.0178 (3)	
C8	−0.0198 (3)	−0.20704 (13)	0.64528 (8)	0.0184 (3)	
C9	−0.1905 (4)	−0.19869 (13)	0.69742 (8)	0.0200 (3)	
H9A	−0.2286	−0.1325	0.7148	0.024*	
C10	−0.3050 (4)	−0.28705 (14)	0.72402 (8)	0.0213 (3)	
H10A	−0.4215	−0.2822	0.7595	0.026*	
C11	−0.2449 (4)	−0.38172 (14)	0.69751 (8)	0.0194 (3)	
C12	−0.0747 (4)	−0.39293 (14)	0.64586 (9)	0.0231 (4)	
H12A	−0.0373	−0.4593	0.6287	0.028*	
C13	0.0391 (4)	−0.30400 (14)	0.62015 (8)	0.0222 (3)	
H13A	0.1581	−0.3094	0.5851	0.027*	

### Atomic displacement parameters (Å^2^)

**Table:**

	*U*^11^	*U*^22^	*U*^33^	*U*^12^	*U*^13^	*U*^23^
Br	0.03917 (15)	0.01765 (14)	0.03289 (15)	−0.00443 (7)	0.01082 (9)	0.00390 (6)
O1	0.0192 (6)	0.0241 (6)	0.0322 (7)	0.0024 (5)	0.0095 (5)	0.0045 (5)
O2	0.0452 (8)	0.0239 (7)	0.0383 (8)	−0.0015 (6)	0.0233 (6)	0.0050 (6)
O3	0.0576 (10)	0.0158 (7)	0.0431 (8)	0.0013 (6)	0.0210 (7)	0.0009 (6)
N1	0.0158 (6)	0.0194 (7)	0.0264 (7)	−0.0010 (5)	0.0077 (5)	0.0028 (6)
N2	0.0279 (7)	0.0197 (8)	0.0222 (7)	0.0006 (6)	0.0048 (6)	0.0032 (6)
C1	0.0165 (7)	0.0188 (8)	0.0194 (8)	−0.0016 (6)	0.0019 (6)	0.0027 (6)
C2	0.0211 (8)	0.0190 (9)	0.0212 (8)	−0.0009 (6)	0.0065 (6)	−0.0020 (6)
C3	0.0235 (8)	0.0211 (9)	0.0223 (8)	−0.0021 (6)	0.0077 (6)	0.0008 (7)
C4	0.0219 (8)	0.0152 (8)	0.0224 (8)	−0.0030 (6)	0.0025 (6)	0.0049 (6)
C5	0.0289 (8)	0.0199 (9)	0.0241 (8)	0.0023 (7)	0.0079 (7)	0.0008 (7)
C6	0.0245 (8)	0.0231 (9)	0.0233 (8)	0.0029 (7)	0.0110 (6)	0.0028 (7)
C7	0.0153 (7)	0.0199 (8)	0.0184 (8)	−0.0010 (6)	0.0026 (6)	0.0008 (6)
C8	0.0153 (7)	0.0202 (8)	0.0194 (7)	0.0006 (6)	0.0012 (6)	0.0028 (6)
C9	0.0232 (8)	0.0157 (8)	0.0217 (8)	0.0019 (6)	0.0050 (6)	−0.0005 (6)
C10	0.0236 (8)	0.0209 (9)	0.0203 (8)	0.0019 (6)	0.0065 (6)	0.0025 (6)
C11	0.0216 (8)	0.0171 (8)	0.0196 (8)	0.0000 (6)	0.0022 (6)	0.0035 (6)
C12	0.0284 (9)	0.0174 (8)	0.0244 (8)	0.0025 (7)	0.0075 (7)	−0.0014 (6)
C13	0.0240 (8)	0.0220 (9)	0.0220 (8)	0.0011 (6)	0.0091 (6)	0.0006 (7)

### Geometric parameters (Å, °)

**Table:**

Br—C4	1.9002 (17)	C5—C6	1.390 (3)
O1—C7	1.223 (2)	C5—H5A	0.9500
O2—N2	1.226 (2)	C6—H6A	0.9500
O3—N2	1.217 (2)	C7—C8	1.504 (2)
N1—C7	1.357 (2)	C8—C13	1.393 (2)
N1—C1	1.418 (2)	C8—C9	1.396 (2)
N1—H1A	0.8800	C9—C10	1.392 (2)
N2—C11	1.475 (2)	C9—H9A	0.9500
C1—C2	1.395 (2)	C10—C11	1.379 (3)
C1—C6	1.396 (3)	C10—H10A	0.9500
C2—C3	1.389 (2)	C11—C12	1.389 (3)
C2—H2A	0.9500	C12—C13	1.389 (3)
C3—C4	1.387 (3)	C12—H12A	0.9500
C3—H3A	0.9500	C13—H13A	0.9500
C4—C5	1.385 (2)		
			
C7—N1—C1	125.41 (14)	C1—C6—H6A	119.6
C7—N1—H1A	117.3	O1—C7—N1	124.04 (16)
C1—N1—H1A	117.3	O1—C7—C8	120.64 (16)
O3—N2—O2	123.49 (16)	N1—C7—C8	115.31 (14)
O3—N2—C11	118.72 (15)	C13—C8—C9	120.04 (16)
O2—N2—C11	117.79 (15)	C13—C8—C7	118.42 (15)
C2—C1—C6	119.53 (16)	C9—C8—C7	121.52 (15)
C2—C1—N1	122.74 (16)	C10—C9—C8	120.17 (16)
C6—C1—N1	117.71 (15)	C10—C9—H9A	119.9
C3—C2—C1	120.10 (16)	C8—C9—H9A	119.9
C3—C2—H2A	120.0	C11—C10—C9	118.27 (16)
C1—C2—H2A	120.0	C11—C10—H10A	120.9
C4—C3—C2	119.30 (15)	C9—C10—H10A	120.9
C4—C3—H3A	120.3	C10—C11—C12	123.09 (16)
C2—C3—H3A	120.3	C10—C11—N2	118.11 (15)
C5—C4—C3	121.64 (16)	C12—C11—N2	118.80 (16)
C5—C4—Br	119.13 (13)	C13—C12—C11	117.87 (17)
C3—C4—Br	119.23 (13)	C13—C12—H12A	121.1
C4—C5—C6	118.67 (16)	C11—C12—H12A	121.1
C4—C5—H5A	120.7	C12—C13—C8	120.55 (16)
C6—C5—H5A	120.7	C12—C13—H13A	119.7
C5—C6—C1	120.71 (16)	C8—C13—H13A	119.7
C5—C6—H6A	119.6		
			
C7—N1—C1—C2	31.0 (3)	O1—C7—C8—C9	146.78 (17)
C7—N1—C1—C6	−150.50 (17)	N1—C7—C8—C9	−32.2 (2)
C6—C1—C2—C3	2.0 (3)	C13—C8—C9—C10	−0.7 (2)
N1—C1—C2—C3	−179.60 (15)	C7—C8—C9—C10	−178.88 (15)
C1—C2—C3—C4	−0.3 (3)	C8—C9—C10—C11	0.0 (2)
C2—C3—C4—C5	−1.3 (3)	C9—C10—C11—C12	0.3 (3)
C2—C3—C4—Br	178.45 (13)	C9—C10—C11—N2	−179.98 (15)
C3—C4—C5—C6	1.3 (3)	O3—N2—C11—C10	−173.01 (17)
Br—C4—C5—C6	−178.45 (13)	O2—N2—C11—C10	6.7 (2)
C4—C5—C6—C1	0.3 (3)	O3—N2—C11—C12	6.7 (2)
C2—C1—C6—C5	−2.0 (3)	O2—N2—C11—C12	−173.58 (16)
N1—C1—C6—C5	179.52 (16)	C10—C11—C12—C13	0.0 (3)
C1—N1—C7—O1	−3.9 (3)	N2—C11—C12—C13	−179.68 (15)
C1—N1—C7—C8	175.02 (14)	C11—C12—C13—C8	−0.7 (3)
O1—C7—C8—C13	−31.4 (2)	C9—C8—C13—C12	1.0 (2)
N1—C7—C8—C13	149.60 (16)	C7—C8—C13—C12	179.28 (15)

### Hydrogen-bond geometry (Å, °)

**Table:**

*D*—H···*A*	*D*—H	H···*A*	*D*···*A*	*D*—H···*A*
N1—H1A···O1^i^	0.88	2.33	3.0026 (18)	133
N1—H1A···O2^ii^	0.88	2.59	3.284 (2)	136
C3—H3A···O1^iii^	0.95	2.45	3.284 (2)	146
C5—H5A···O3^iv^	0.95	2.52	3.447 (2)	166
C6—H6A···O2^ii^	0.95	2.49	3.354 (2)	151
C9—H9A···O2^ii^	0.95	2.48	3.397 (2)	162

Symmetry codes: (i) *x*−1, *y*, *z*; (ii) −*x*−1, *y*+1/2, −*z*+3/2; (iii) −*x*+1, −*y*, −*z*+1; (iv) *x*, *y*+1, *z*.
